# Pop2 phosphorylation at S39 contributes to the glucose repression of stress response genes, *HSP12* and *HSP26*

**DOI:** 10.1371/journal.pone.0215064

**Published:** 2019-04-11

**Authors:** Pham Thi Kim Lien, Nguyen Thi Minh Viet, Tomoaki Mizuno, Yasuyuki Suda, Kenji Irie

**Affiliations:** 1 Department of Molecular Cell Biology, Graduate School of Comprehensive Human Sciences and Faculty of Medicine, University of Tsukuba, Tsukuba, Japan; 2 VN-UK Institute for Research and Executive Education, The University of Danang, Danang, Vietnam; 3 Live Cell Super-resolution Imaging Research Team, RIKEN Center for Advanced Photonics, Wako, Saitama, Japan; Kindai University, JAPAN

## Abstract

The *S*. *cerevisiae* Pop2 protein is an exonuclease in the Ccr4-Not complex that is a conserved regulator of gene expression. Pop2 regulates gene expression post-transcriptionally by shortening the poly(A) tail of mRNA. A previous study has shown that Pop2 is phosphorylated at threonine 97 (T97) by Yak1 protein kinase in response to glucose limitation. However, the physiological importance of Pop2 phosphorylation remains unknown. In this study, we found that Pop2 is phosphorylated at serine 39 (S39) under unstressed conditions. The dephosphorylation of S39 was occurred rapidly after glucose depletion, and the addition of glucose to the glucose-deprived culture recovered this phosphorylation, suggesting that Pop2 phosphorylation at S39 is regulated by glucose. This glucose-regulated phosphorylation of Pop2 at S39 is dependent on Pho85 kinase. We previously reported that Pop2 takes a part in the cell wall integrity pathway by regulating *LRG1* mRNA; however, S39 phosphorylation of Pop2 is not involved in *LRG1* expression. On the other hand, Pop2 phosphorylation at S39 is involved in the expression of *HSP12* and *HSP26*, which encode a small heat shock protein. In the medium supplemented with glucose, Pop2 might be phosphorylated at S39 by Pho85 kinase, and this phosphorylation contributes to repress the expression of *HSP12* and *HSP26*. Glucose starvation inactivated Pho85, which resulted in the derepression of *HSP12* and *HSP26*, together with other glucose sensing mechanisms. Our results suggest that Pho85-dependent phosphorylation of Pop2 is a part of the glucose sensing system in yeast.

## Introduction

Post-transcriptional regulation, including the control of mRNA degradation and translation, plays important roles in regulation of gene expression. mRNA degradation is generally initiated by poly(A) tail shortening referred to as deadenylation, which is catalyzed by the Ccr4-Not and the Pan2-Pan3 complexes [1]. In *Saccharomyces cerevisiae*, the Ccr4-Not complex consists of 9 core subunits, including Ccr4, Pop2/Caf1, Caf40, Caf130 and Not1-5, all of which have homologs in most other eukaryotes [2]. Ccr4 and Pop2 are two active 3’ to 5’ exonucleases: Ccr4 is the major one; Pop2 may modulate specific mRNA expression in certain conditions [1]. Pop2 is reportedly involved in expression control of glucose-repressed genes and regulation of the cell wall integrity (CWI) pathway in *S*. *cerevisiae* [3, 4]. The loss of *POP2* results in pleiotropic phenotypes including temperature-sensitive growth, abnormal cell morphology, weak cell lysis and defective glucose repression [3, 5]. Pop2 is phosphorylated by Yak1 kinase at threonine 97 (T97) in response to a glucose-regulating signal [6]. This modification is required for cell growth control and cell cycle arrest at G1 phase after glucose starvation [6]. We have previously shown that Pop2 functions in the CWI pathway by regulating the expression of *LRG1*, encoding a GTPase-activating protein for Rho1 small GTPase [4]. The *LRG1* mRNA level is elevated in *pop2*Δ and the high temperature-sensitivity phenotype caused by *pop2*Δ mutation is suppressed by deletion of *LRG1* [4].

In the budding yeast, Pho85 is a multifunctional cyclin-dependent protein kinase (CDK), which was originally identified as a negative regulator of *PHO5* expression through the control of a transcription factor Pho4 [7, 8]. Pho85 is regulated by the association with ten cyclins, Pho80 and Pcls, and involved in multiple cellular processes beyond the important roles in cell cycle control: for example, metabolism of nutrients such as phosphate and carbon sources [9, 10]. The *pho85*Δ mutant shows pleiotropic phenotypes, including the slow growth with a G1 delay on rich media, a severe growth defect on non-fermentable carbon sources, abnormal morphology, irregular budding and hyperaccumulation of glycogen [9, 11–13]. Additionally, *PHO85* deletion causes the elevated expression not only of phosphate-starvation-dependent genes but also of the stress responsive genes such as *HSP12* and *UBI4* [14, 15]. Thus, Pho85 is activated in satisfactory environmental conditions in order to ensure the turn-off of stress responses and inappropriate activities [16]. However, few Pho85 targets have been unambiguously identified; therefore, a long-time goal is understanding molecular functions and substrates that provide interpretations for each phenotype caused by the *pho85*Δ mutation.

In this study, to investigate how Pop2 is regulated upon glucose availability, we examined the phosphorylation of Pop2 and its function. Our results presented here suggest that Pop2 is phosphorylated at S39 in a Pho85-dependent manner upon glucose availability. Furthermore, this post-transcriptional modification of Pop2 specifically contributes to the glucose repression of the stress response genes, *HSP12* and *HSP26*.

## Materials and methods

### Strains, plasmids and general methods

*Escherichia coli* DH5α was used for DNA manipulations. The present yeast strains and plasmids are described in S1 and S2 Tables. Cells were grown in yeast extract-peptone dextrose (YPD), yeast extract-peptone glycerol lactate (YPGL), synthetic complete medium (SC), and SC media lacking either amino acids or other nutrients (SC–Ura, SC lacking uracil). General procedures were performed as described previously [17].

### Gene deletion and protein tagging

Gene disruption and insertion were performed using PCR-based gene replacement, as described previously [18, 19].

### Western Blot analysis

Cells grown to exponential phase were subjected to a mild alkali treatment-based protein extraction method [20]. Samples were loaded onto SDS-PAGE gel and then electroblotted onto Immobilon polyvinylidene difluoridemembranes (MerckMillipore, USA). Phos-tag (Wako, Japan) was added to a mix of SDS-PAGE gel when required. Blots were blocked for 1 h at room temperature with TBS-M buffer containing 20 mM Tris-HCl (pH 7.5), 150 mM NaCl, and 5% non-fat dry milk, and then incubated with 1:1,000-diluted primary antibodies in TBS-M buffer overnight at 4°C. After three final washes with TBS buffer containing 20 mM Tris-HCl (pH 7.5) and 150 mM NaCl, blots were incubated with secondary antibodies, and were developed using enhanced chemiluminescence detection kits (Merck Millipore, USA). Signal intensities were quantified by Image Studio software (LI-COR).

### Immunoprecipitation of Pop2Flag

Cells were grown in YPD medium at 30°C to exponential phase and harvested by centrifugation. The cells were then re-suspended in XT buffer containing 50 mM HEPES-KOH (pH 7.3), 20 mM potassium acetate, 2 mM EDTA, 0.1% Triton X-100, 5% glycerol protease inhibitors, phenylmethylsulfonyl fluoride (PMSF), aprotinin, and leupeptin. Glass beads were then added and cells were broken by rigorous vortexing at 4°C (4 times at 3,500 rpm for 30 s). Lysates were then centrifuged for 10 min at 15,000 *g* and supernatants were collected.

To immunoprecipitate Pop2Flag, extracts were incubated with anti-Flag antibody coupled to protein G-Sepharose beads (Sigma Aldrich, USA) for 30 min at 4°C. Pop2Flag-bound beads were then washed three times in XT buffer, and the bound material was eluted with elution buffer containing 0.1 mg/mL Flag peptide in XT buffer for 10 min at 4°C. Eluted Pop2Flag samples were split and one-half treated with λ-phosphatase (NEB #P0753S, USA) for 2 h at 30°C. Samples were then subjected to SDS-PAGE with or without phos-tag followed by Immunoblotting with anti-Flag antibody.

### RNA isolation, RT-qPCR, and microarray analysis

Total RNA was isolated using ISOGEN reagent (Nippon Gene, Japan) and the RNeasy Mini kit (Qiagen, Germany). First strands of cDNA were generated using the PrimeScript RT reagent Kit (Takara, Japan). The cDNAs of *LRG1*, *HSP12*, *HSP26*, and *PIR3* were quantitated by a quantitative real-time PCR (qRT-PCR) method using a 7500 fast real-time RT-PCR system (Applied Biosystems) with SYBR Premix Ex Taq (Takara, Japan). A standard curve was generated from diluted cDNA derived from wild-type cells, and levels of gene expression were normalized to *ACT1* expression. The microarray analysis was performed by the KURABO Bio-Medical Department (Japan) using the Affymetrix GeneChip Yeast Genome 2.0 Array (Affymetrix, Santa Clara, Califronia, USA). Microarray data sets are available at the Gene Expression Omnibus at http://www.ncbi.nlm.nih.gov/geo (GEO accession number GSE124908).

### Statistical analysis

Data are presented as means ± standard deviations (SD) of 3 independent experiments. Statistical analyses were performed using analysis of variance (ANOVA) followed by Tukey’s test or student’s t-test test. Differences were considered significant when p < 0.01 or p < 0.05.

## Results

### Pop2 is a phosphorylated protein

**Fig 1:**
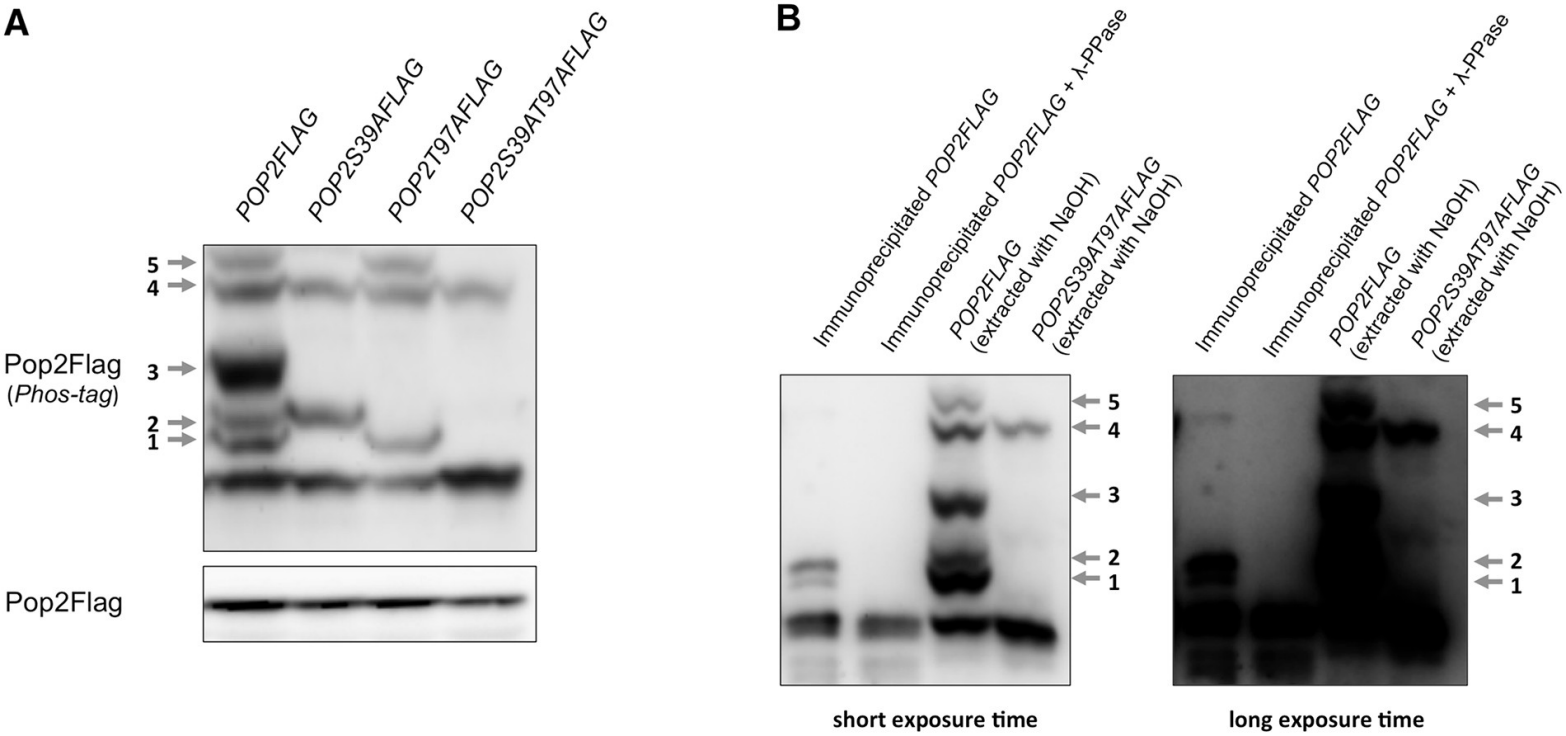
Pop2 is phosphorylated at S39 and T97. (A) *pop2*Δ mutant cells harboring YCplac33-*POP2FLAG* (*POP2FLAG*), YCplac33-*POP2S39AFLAG* (*POP2S39AFLAG)* were grown at 30°C until exponential phase. Extracts prepared from each strain were run on phos-tag and conventional gels, then immunoblotted with anti-Flag antibody. Phosphorylated Pop2Flag is indicated with arrows and numbers according to the positions. Band 3 appears as dominant in the *pop2Δ* mutant harboring the *POP2-FLAG* plasmid, but less dominant in the WT strain harboring the *POP2-FLAG* plasmid (see Fig 2B). (B) Wild-type cells harboring YCplac33-*POP2FLAG* (*POP2FLAG*) were grown at 30°C until exponential phase. Extracts prepared from this strain were immunoprecipitated using anti-Flag antibody. Immunoprecipitated Pop2Flag was treated with/withoutλphosphatase. The band patterns of immunoprecipitated Pop2 are compared with the band patterns of Pop2 and Pop2S39AT97A extracted with NaOH as analyzed in (A).

Protein phosphorylation plays an important role in regulating protein function in response to various stimuli. There are few studies investigating post-translational modifications of the Ccr4-Not subunits and the relevance of such modification for their functions. Therefore, we examined the phosphorylation of Pop2, one of the exonucleases in the Ccr4-Not complex, using phosphate affinity (phos-tag) SDS-PAGE. This technique allows for the detection of phosphorylation by the mobility shift [21]. We observed clear electrophoretic shift bands of Pop2Flag under unstressed conditions (Fig 1A). These bands were numbered from 1 to 5 according to their positions. To confirm that the band shifts are caused by Pop2 phosphorylation, immunoprecipitated Pop2Flag were treated with λ-phosphatases. We found that Pop2 mobility shift is abolished by λ-phosphatase treatment (Fig 1B), suggesting that Pop2 is a phosphoprotein. The difference of band patterns between the immunoprecipitated Pop2 and Pop2 in the NaOH-treated crude extract is due to the difference in cell extraction.

Previous global phosphorylation analyses revealed the Serine 39 residue (S39) of Pop2 as a potential phosphorylation site [22–24]. It also has been proposed that Pop2 is phosphorylated at Threonine 97 (T97) [6]. Therefore, we investigated whether Pop2 is phosphorylated at S39 and T97 in a *pop2Δ* background. We constructed a strain expressing Pop2 with phospho-defective mutations at either S39 (Pop2S39A), T97 (Pop2T97A), or both (Pop2S39AT97A), and then examined the mobility of Pop2. Interestingly, the shifted-bands 1, 3, and 5 of Pop2 were lost by S39A mutation, while shifted-bands 2 and 3 were lost by T97A mutation (Fig 1A). Consistently, S39AT97A caused the disappearance of shifted-band 1, 2, 3, and 5 (Fig 1A). Our data suggest that Pop2 is phosphorylated at both S39 and T97. Furthermore, the shifted-band 3 was vanished by either S39A or T97A mutation, providing strong evidence that this band is a result of S39 and T97 phosphorylation combination.

### Pop2 phosphorylation is regulated by glucose availability

**Fig 2:**
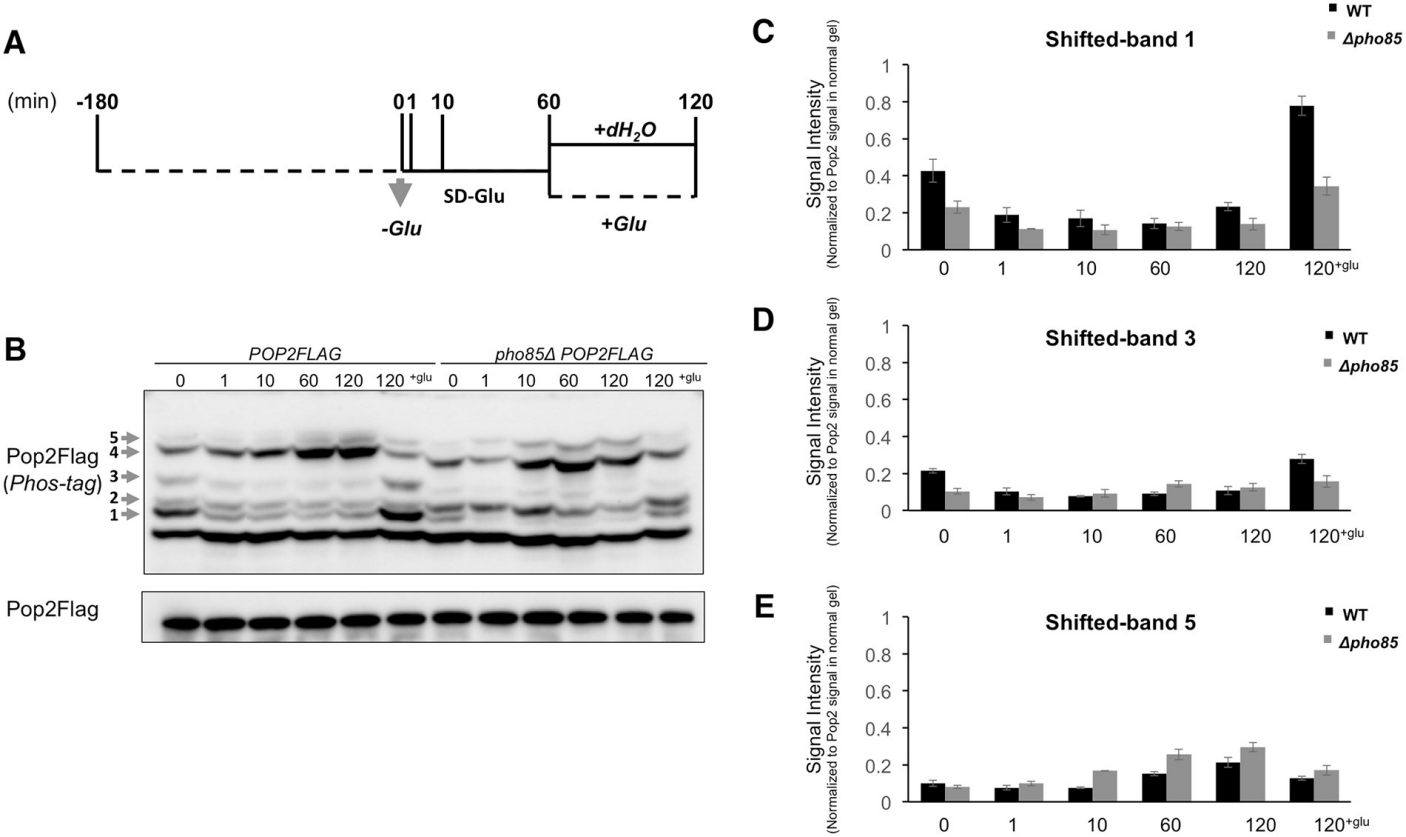
The S39 phosphorylation of Pop2 is regulated by glucose. (A) Schematic illustration of cell collection. Cells were grown at 30°C until exponential phase, washed once with medium lacking glucose and re-suspended in medium lacking glucose. Glucose was then added to the culture to a final concentration of 2%. Cells were collected at indicated times. (B) Wild-type cells and *pho85Δ* mutant cells harboring YCplac33-*POP2FLAG* were collected as in (A). Extracts prepared from each strain were run on phos-tag and conventional gels, then immunoblotted with anti-Flag antibody. Phosphorylated Pop2Flag is indicated with arrows and numbers according to the positions. Representative data are shown. (C, D, E) The intensities of Pop2Flag shifted-band 1, 3, and 5 signals in phos-tag gel were measured and normalized to the Pop2Flag signals in normal gel.

Recent data suggested that the Ccr4-Not complex is involved in glucose-regulating pathways [25]. Therefore, we examined Pop2 phosphorylation in cells cultured transiently in media with and without glucose (Fig 2A). Phosphorylated forms of Pop2 showing in shifted-band 1 and 3 were diminished within 1 min after glucose starvation. Adding glucose to the glucose-deprived culture recovered these bands (Fig 2B, 2C and 2D). On the other hand, the shifted-band 5 was increased upon glucose limitation and decreased after glucose re-addition (Fig 2E). Remarkably, S39A mutation caused the disappearance of shifted-band 1,3, and 5 in the presence of glucose (Fig 1), indicating that phosphorylation of Pop2 at S39 is regulated by glucose availability.

### The S39 phosphorylation regulates Pop2 function to repress the expression of *HSP12* and *HSP26*

**Fig 3:**
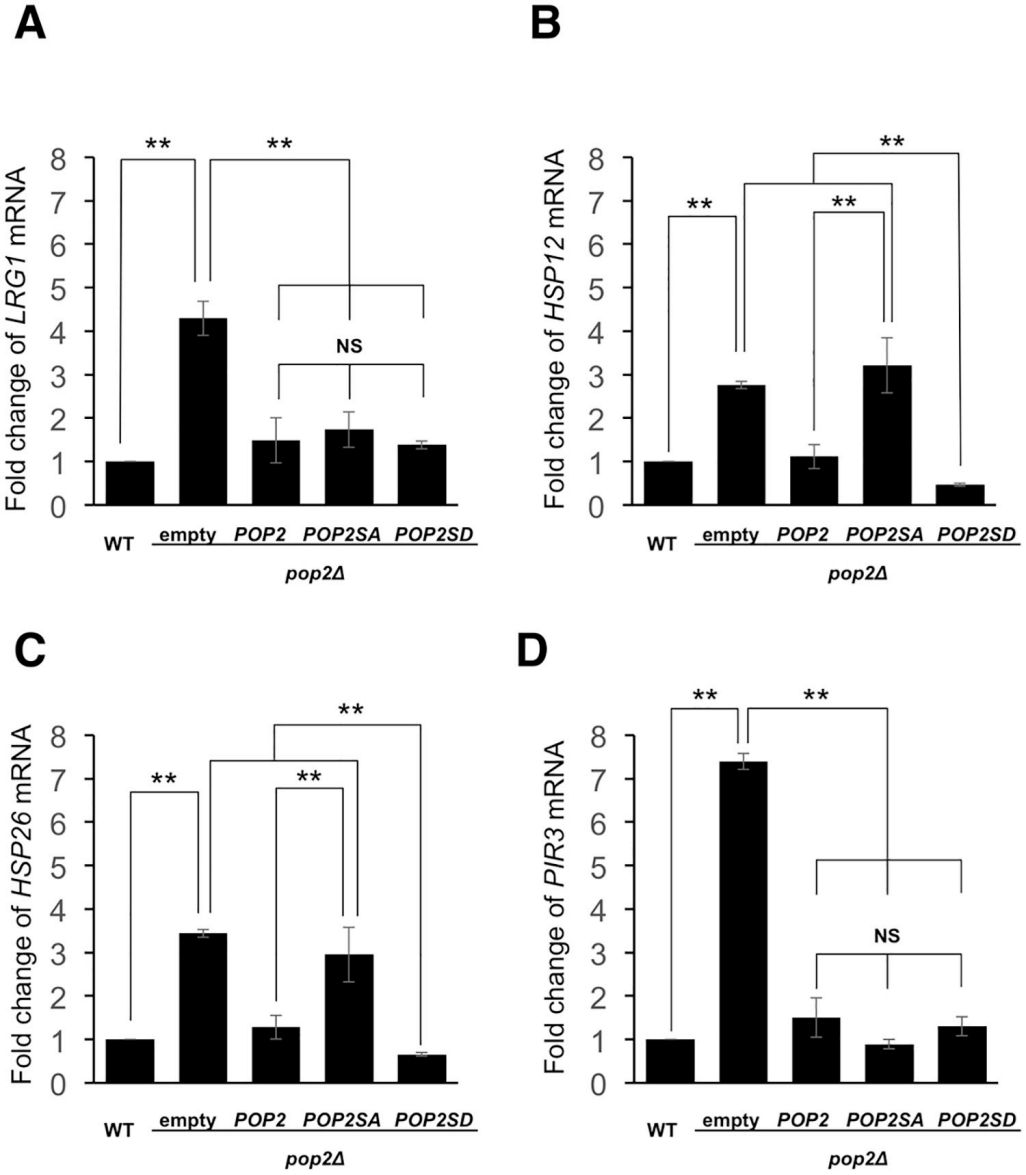
Effect of the S39 phosphorylation on the *LRG1*, *HSP12*, *HSP26* and *PIR3* expression in glucose-containing medium. (A, B, C, D) Expression of *LRG1*, *HSP12*, *HSP26*, *PIR3* mRNAs in WT harboring YCplac33 (empty vector) and *pop2Δ* harboring YCplac33, YCplac33-*POP2FLAG*, YCplac33-*POP2S39AFLAG*, YCplac33-*POP2S39DFLAG*. The cells were grown at 30°C until exponential phase and collected for RNA isolation. mRNA levels were quantified by qRT-PCR. The relative mRNA levels were calculated using delta delta Ct method normalized to *ACT1* reference gene. The data show mean ± the standard deviation (n = 3). NS, not significant, **P < 0.01 as determined by Tukey’s test.

We previously reported that *LRG1* mRNA level is upregulated by *pop2* deletion [4]. Therefore, we examined the effect of S39 phosphorylation on *LRG1* expression. The *pop2* deletion caused increased *LRG1* expression, which was efficiently suppressed by both wild-type Pop2 and Pop2S39A (Fig 3A). Furthermore, Pop2 with a phospho-mimetic mutation at S39 (S39D) also efficiently suppressed the increased *LRG1* expression caused by *pop2Δ* mutation (Fig 3A). This result suggests that S39 phosphorylation is unrelated to the regulation of *LRG1* mRNA levels.

To identify other Pop2 targets, we measured the gene expression levels in wild-type and *pop2Δ* mutant cells by carrying out a microarray analysis. Our microarray data showed that *pop2* deletion upregulated expression of many genes involved in stress responses, such as *HSP12*, *HSP26*, and *PIR3* (S3 Table). To confirm the microarray data, we examined the mRNA levels of *HSP12*, *HSP26*, and *PIR*3 in wild-type and *pop2Δ* mutant cells by real time RT-PCR. These mRNA levels were increased by *pop2Δ* mutation, indicating that Pop2 functions in glucose repression of *HSP12*, *HSP26* and *PIR3* (Fig 3B, 3C and 3D). We next asked whether S39 phosphorylation affects the ability of Pop2 to repress the expression of *HSP12*, *HSP26*, and *PIR3*. We found that wild-type *POP2* efficiently complemented the increased expression of *HSP12* and *HSP26* caused by *pop2Δ* mutation, but *POP2-S39A* did not (Fig 3B and 3C). Additionally, *POP2-S39D* also efficiently complemented the increased expression of *HSP12* and *HSP2*6 (Fig 3B and 3C). In contrast, introduction of Pop2S39A and Pop2S39D as well as wild-type Pop2 fully recovered the increased expression of *PIR3* observed in *pop2Δ* mutant cells (Fig 3D). Thus, S39 phosphorylation is involved in the regulation of *HSP12* and *HSP26*, but not in that of *PIR3*.

**Fig 4:**
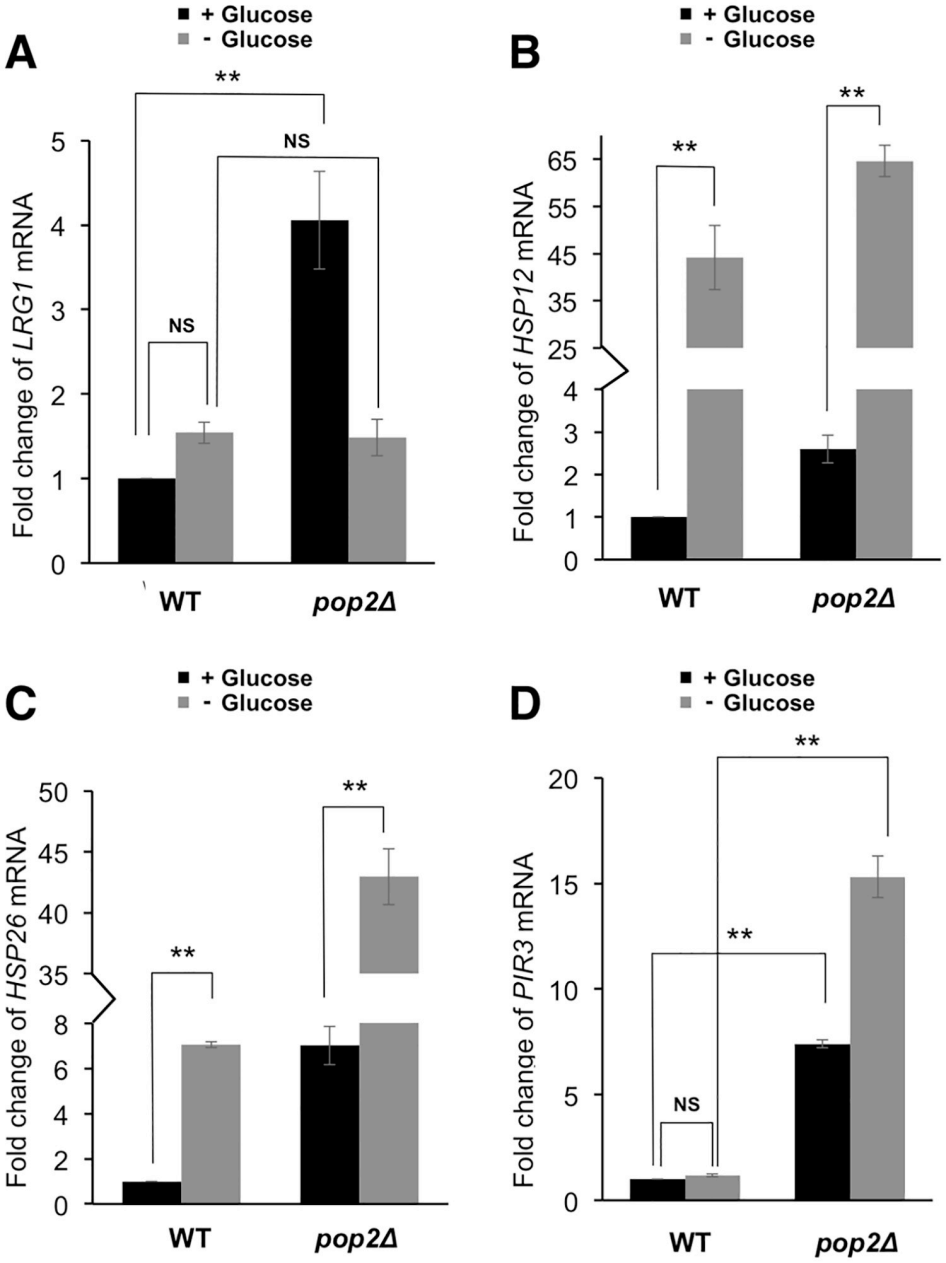
The expression of *LRG1*, *HSP12*, *HSP26* and *PIR3* in wild-type and *pop2Δ* in glucose and glucose-deprived conditions. (A, B, C, D) Expression of *LRG1*, *HSP12*, *HSP26*, *PIR3* mRNAs in wild-type cells and *pop2Δ* mutant cells. mRNA levels were quantified by qRT-PCR. Wild-type, *pop2Δ* cells were grown at 30°C until exponential phase, washed once with medium lacking glucose and re-suspended in medium lacking glucose for 1 h. Cells were collected before and after glucose starvation for RNA isolation. The relative mRNA levels were calculated using delta delta Ct method normalized to *ACT1* reference gene. The data show mean ± the standard deviation (n = 3). NS, not significant, **P < 0.01 as determined by Tukey’s test.

Since the phosphorylation of Pop2 at S39 is regulated by glucose availability as described above (Fig 2), we next examined whether expression of *HSP12* and *HSP26* are affected by glucose availability. The levels of *HSP12* and *HSP26* mRNAs were upregulated by glucose starvation (Fig 4B and 4C). On the other hand, the expression of *LRG1* and *PIR3* was similar in the presence and absence of glucose (Fig 4A and 4D). These data are consistent with the idea that the phosphorylation of Pop2 at S39 in glucose medium is involved in the glucose repression of *HSP12* and *HSP26*. Since the mRNA levels of *HSP12* and *HSP26* in *pop2Δ* mutant cells were further upregulated by glucose starvation (Fig 4B and 4C), the *POP2*-independent mechanisms should be involved in the glucose repression of *HSP12* and *HSP26*. The mRNA levels of *HSP12* and *HSP26* in *POP2-S39A* and *POP2-S39D* cells were also upregulated by glucose starvation (data not shown). The *POP2*-independent mechanisms include Hsf1 and Msn2/4 transcription factors [26–28], which are involved in multiple glucose sensing pathways in yeast [29]. Although the effect of the *POP2-S39A* mutation is much smaller than the effect of glucose depletion (3-fold in Fig 3B vs 45-fold in Fig 4B), Pop2 phosphorylation still contributes to glucose repression of *HSP12* and *HSP26* in some extent. Our results also showed that the impacts of Pop2 regulation on gene expression are different between *LRG1* and *PIR3*. *LRG1* mRNA might be degraded in a *POP2*-dependent manner in glucose medium but not in glucose starvation, while expression of *PIR3* is dependent on *POP2* in both conditions (Fig 4A and 4D).

### Pop2 might be phosphorylated by Pho85

The amino acid sequence surrounding S39 of Pop2 is matched to the phosphorylation consensus sequence of CDK substrates, [(K)-S/T-P-X-(K/R)], in which S/T is a phosphorylation site [30]. In the PhosphoPep database (http://www.phosphopep.org/), Pop2 phosphorylation at S39 is shown to be dependent on Pho85 [31]. Therefore, we examined Pop2 phosphorylation in the *pho85Δ* mutant. The signals of the shifted-bands numbered 1 and 3, corresponding to S39 phosphorylation (Fig 1), were substantially reduced and almost disappeared in *pho85Δ* (Fig 2B). These data support the idea that Pop2 is phosphorylated at S39 by Pho85. *PHO85* is a homolog of *CDC28* [32]. Therefore, we also examined Pop2 phosphorylation in temperature-sensitive *cdc28* mutants, *cdc28-4* and *cdc28-13*. However, Pop2 phosphorylation was not altered in these mutants even at non-permissive temperature (data not shown), suggesting that Cdc28 is not involved in S39 phosphorylation.

**Fig 5:**
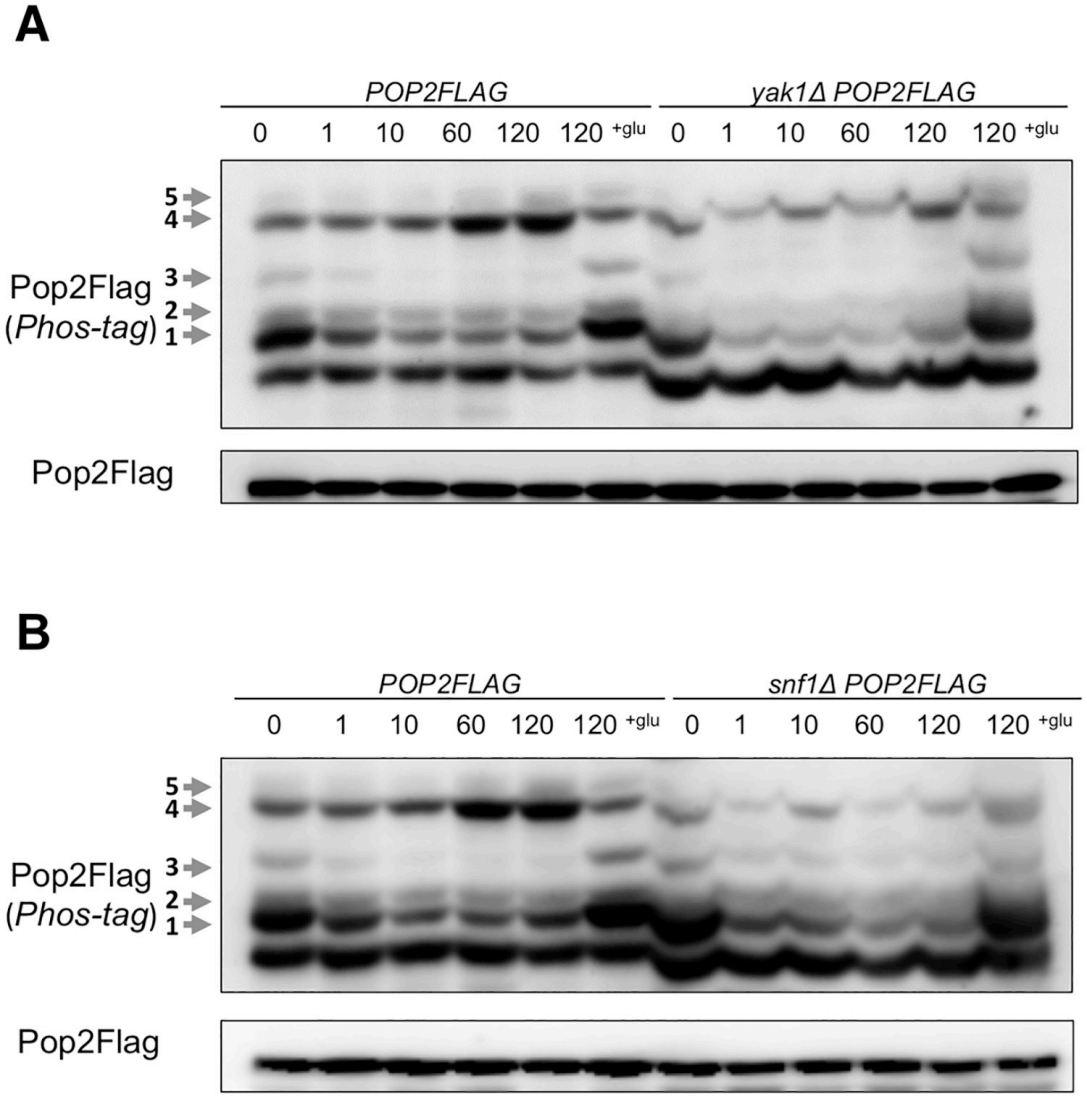
The phosphorylation of Pop2 in *yak1Δ* and *snf1Δ*. (A) WT and *snf1Δ* cells harboring YCplac33-*POP2FLAG* (*POP2FLAG*, *snf1ΔPOP2FLAG*) were collected as in Fig 2A. Extracts prepared from each strain were run on phos-tag and conventional gels, then immunoblotted with anti-Flag antibody. Phosphorylated Pop2Flag is indicated with arrows and numbers according to the positions. Representative data are shown. (B) WT and *yak1Δ* cells harboring YCplac33-*POP2FLAG* (*POP2FLAG*, *yak1ΔPOP2FLAG*) were collected as in Fig 2A. Samples were analyzed as in (A). Representative data are shown.

We also found that glucose deprivation-mediated changes of shifted-band 1 and shifted-band 3 were hardly observed in *pho85Δ* (Fig 2B), but still observed in *yak1Δ* and *snf1Δ* cells (Fig 5A and 5B). Thus, Yak1 and Snf1 may not be involved in generation of these two phosphorylation forms of Pop2. Taken together, our results suggest that phosphorylation of Pop2 at S39 is dependent on Pho85 kinase.

### Expression of *HSP12* and *HSP26* is upregulated by *pho8*5 deletion in glucose condition

**Fig 6:**
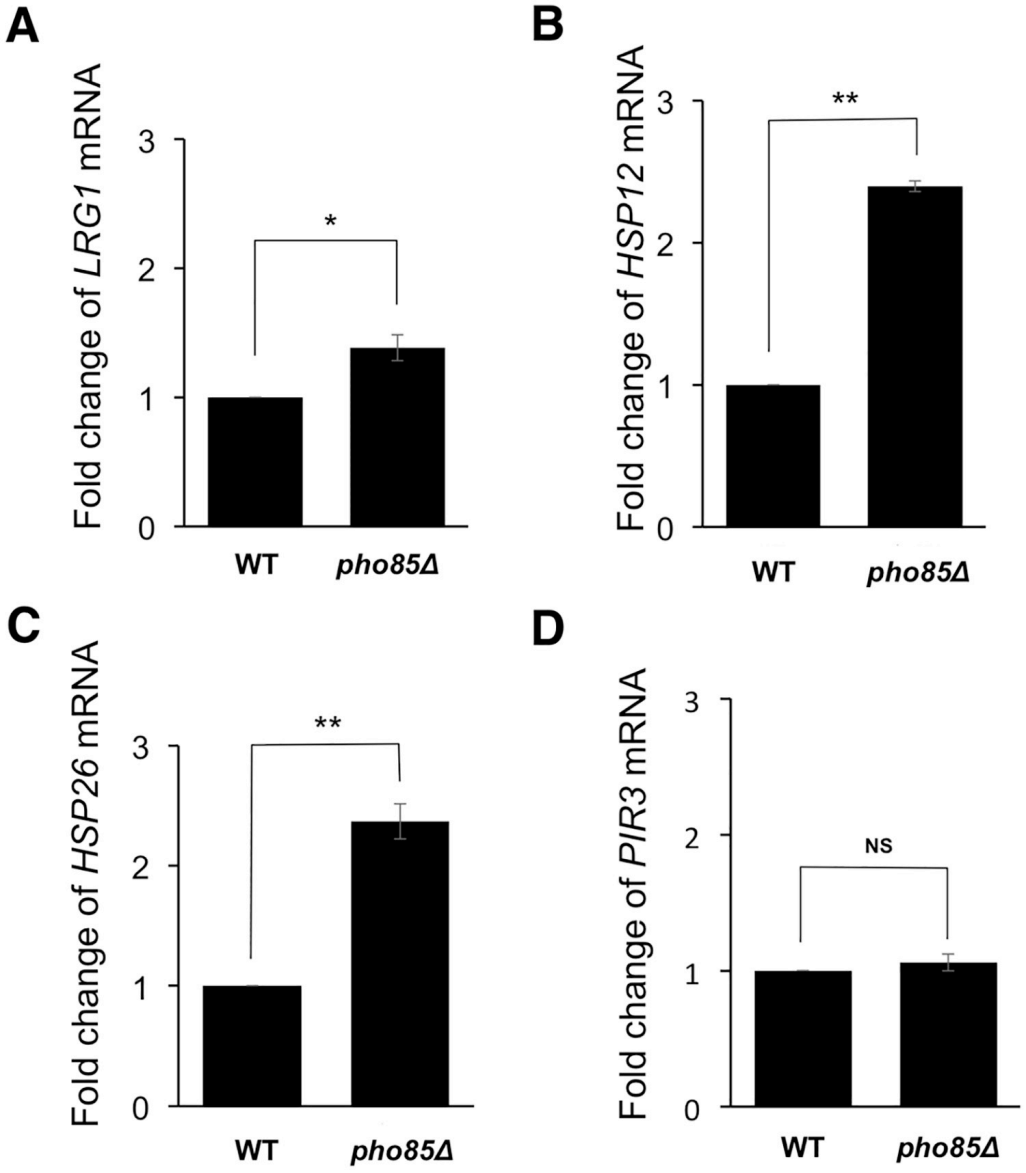
Expression of *HSP12* and *HSP26* is elevated by *PHO85* deletion in glucose medium. (A, B, C, D) Expression of *LRG1*, *HSP12*, *HSP26*, *PIR3* mRNAs in wild-type cells and *pho85Δ* mutant cells. mRNA levels were quantified by qRT-PCR. WT, *pho85Δ* cells were grown at 30°C until exponential phase. The cells were collected for RNA isolation. The relative mRNA levels were calculated using delta delta Ct method normalized to *ACT1* reference gene. The data show mean ± the standard deviation (n = 3). NS, not significant, *P < 0.05, **P < 0.01 as determined by student’s t-test.

**Fig 7:**
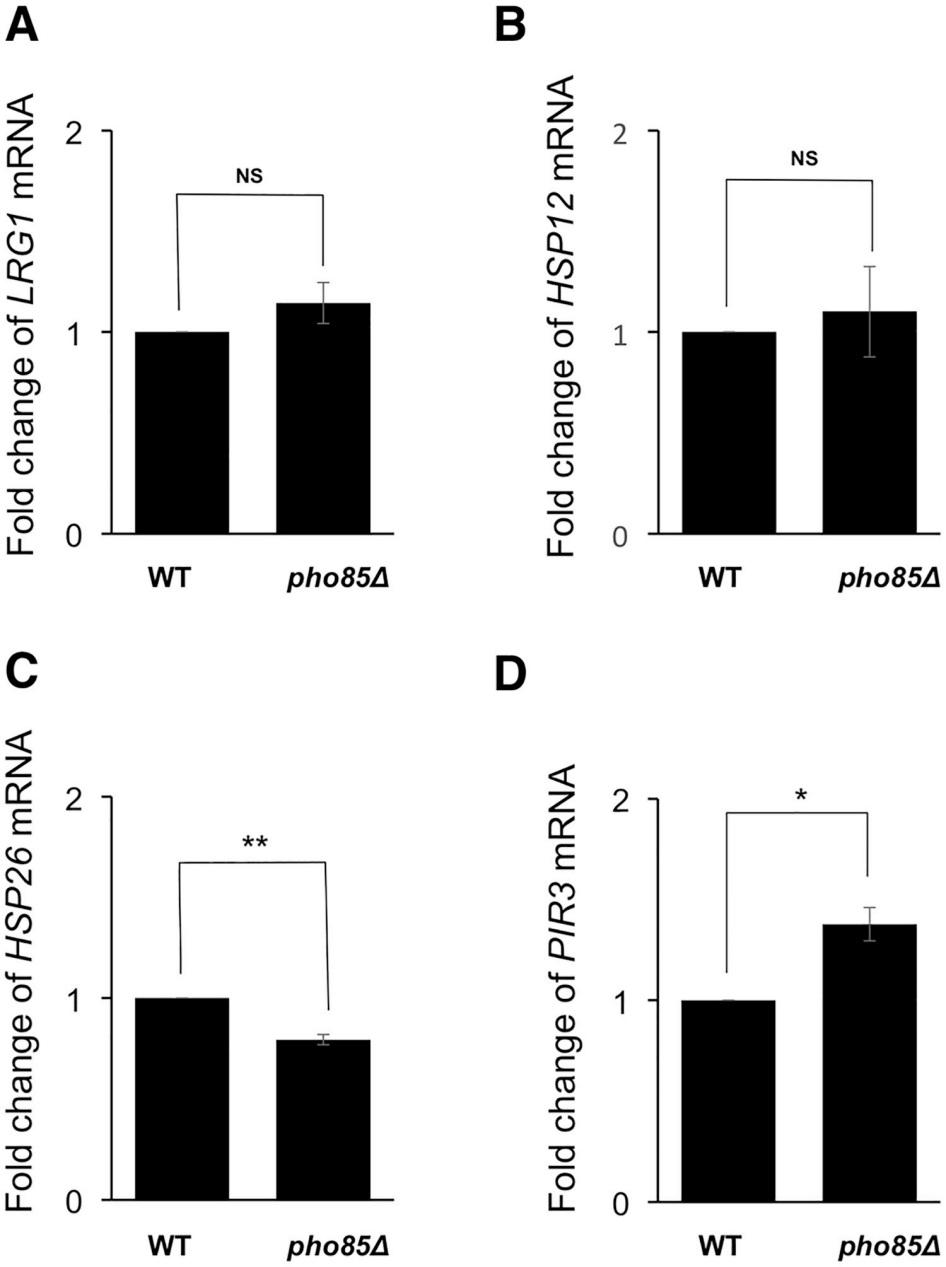
Expression of *LRG1*, *HSP12*, *HSP26* and *PIR3* is not significantly affected by *PHO85* deletion in glucose starvation. (A, B, C, D) Expression of *LRG1*, *HSP12*, *HSP26*, *PIR3* mRNAs in wild-type cells and *pho85Δ* mutant cells. mRNA levels were quantified by qRT-PCR. WT, *pho85Δ* cells were grown at 30°C until exponential phase, washed once with medium lacking glucose and re-suspended in medium lacking glucose for 1 h. The cells were collected after glucose starvation for RNA isolation. The relative mRNA levels were calculated using delta delta Ct method normalized to *ACT1* reference gene. The data show mean ± the standard deviation (n = 3). NS, not significant, *P < 0.05, **P < 0.01 as determined by student’s t-test.

Pho85 kinase functions in the suppression of stress response genes under glucose-rich conditions [15]. Therefore, we examined the effect of Pho85 on *LRG1*, *HSP12*, *HSP26*, and *PIR3* expression in the medium supplemented with glucose. As shown in Fig 6B and 6C, *HSP12* and *HSP26* expression in *pho85Δ* cells were more than 2-fold higher than wild-type cells. On the other hand, *pho85* deletion slightly increased and did not change the *LRG1* mRNA and *PIR3* mRNA levels, respectively (Fig 6A and 6D). Moreover, *pho85* deletion did not significantly increase *HSP12* and *HSP26* expression when the cells were moved to glucose starvation conditions (Fig 7). Our data suggest that Pho85 partly contributes to repress *HSP12* and *HSP26* expression, probably through the phosphorylation of Pop2 at S39 in glucose medium.

## Discussion

### Multiple migration forms of Pop2

In this study, we showed that Pop2 is phosphorylated at S39 in a Pho85 kinase-dependent manner in the normal growth condition. Our results suggest that three phosphorylated forms of Pop2, shifted-band 1, 3, and 5, are dependent on S39 (Fig 1), while only two phosphorylated forms, shifted-band 1 and 3, are dependent on *PHO85* (Fig 2B, 2C and 2D). A previous study showed that Pop2 is phosphorylated at T97 by Yak1 in response to glucose limitation [6]. Consistently, the shifted-band 2 seen in our phos-tag SDS-PAGE was dependent on T97 and *YAK1*: the shifted-band 2 was disappeared by T97A mutation (Fig 1A) and in *yak1Δ* mutant (Fig 5A). Either S39A or T97A mutation of *POP2* leads to the loss of shifted-band 3, suggesting that this band is derived from Pop2 phosphorylated at both S39 and T97. We also found that the shifted-band 4 and 5 were significantly increased upon glucose depletion in wild-type cells (Fig 2B), but not in *snf1Δ* and *yak1Δ* mutants (Fig 5A and 5B). Thus, these two bands are dependent on Snf1 and Yak1. Furthermore, S39A also causes the loss of shifted-band 5 (Fig 1A); therefore, the shifted-band 5 likely results from Pop2 phosphorylated not only at S39 but also at other unknown sites. Thus, Pop2 is phosphorylated and regulated by multiple kinases.

### Glucose-dependent phosphorylation of Pop2-S39

When exponentially growing cells were transferred to the glucose-deprived medium, the shifted-band 1 and 3 of Pop2, which correspond with S39 phosphorylation, rapidly disappeared (Fig 2B, 2C and 2D). After glucose addition to the glucose-starved culture, those phosphorylated forms were recovered. The signals of shifted-band 1 and 3 were substantially decreased but not completely disappeared in the *pho85Δ* mutant. Additionally, the shifted-band 5, which is also dependent on S39 phosphorylation (Fig 1A), was increased upon glucose starvation in wild-type as well as *pho85Δ* (Fig 2B and 2E). This suggests that the phosphorylation of Pop2 at S39 is dependent not only on Pho85 but also on other kinases, which might be glucose-activated Snf1 and Yak1 (Fig 5A and 5B). S39 phosphorylation dependent on Pho85 is decreased upon glucose depletion and reappeared upon glucose addition (Fig 2B, 2C and 2D). Furthermore, *pho85Δ* mutation has almost no effect on the expression of *HSP12* and *HSP26* in glucose starvation (Fig 7), suggesting that Pho85 kinase activity is regulated by glucose availability. Previous studies showed that Pho85 has multiple cyclin partners, which determine the substrate specificity [16]. Investigating whether Pho85 and its cyclin partners directly phosphorylate Pop2 will be the focus of future studies.

### S39-phosphorylated Pop2 contributes to glucose repression of *HSP12* and *HSP26*

Our previous studies showed that Pop2, together with Ccr4, Dhh1, and Puf5, is involved in regulation of *LRG1* expression [4, 33, 34]. This regulation is mediated by mRNA stability control. S39 phosphorylation of Pop2 is not involved in regulation of *LRG1* expression, since *POP2-S39A* efficiently suppressed the increased level of *LRG1* mRNA caused by *pop2Δ* mutation (Fig 3A). We searched other targets for Pop2 and then examined whether Pop2 phosphorylation at S39 is involved in their expression. We found that Pop2 phosphorylation at S39 specifically regulates Pop2 function to repress the expression of certain stress response genes, *HSP12* and *HSP26* (Fig 3B and 3C). In the *pop2Δ* mutant, these mRNA levels are increased in glucose-containing media, indicating that Pop2 is involved in glucose repression of *HSP12* and *HSP26*. Wild-type *POP2* efficiently complemented the increased expression of *HSP12* and *HSP26* caused by *pop2Δ* mutation, but *POP2-S39A* did not (Fig 3B and 3C). Interestingly, *POP2-S39A* completely complemented the increased expression of *PIR3* caused by *pop2Δ* mutation (Fig 3D). Thus, the effect of S39A mutation is gene specific. How does Pop2 phosphorylated at S39 regulate the expression of *HSP12* and *HSP26*? Since S39 phosphorylation of Pop2 is not involved in the regulation of *LRG1* expression, it might be unrelated to regulation of Pop2 deadenylase activity. Pop2 was originally found as a regulator of *PGK1* expression [3]. The Ccr4-Not complex is known to regulate gene expression not only through mRNA degradation but also through other mechanisms including transcriptional initiation and elongation [35]. One possibility is that S39 phosphorylation specifically influences the interaction of Pop2 with other factors involved in transcriptional initiation and elongation. Further studies are required to reveal this regulation.

## Conclusions

**Fig 8:**
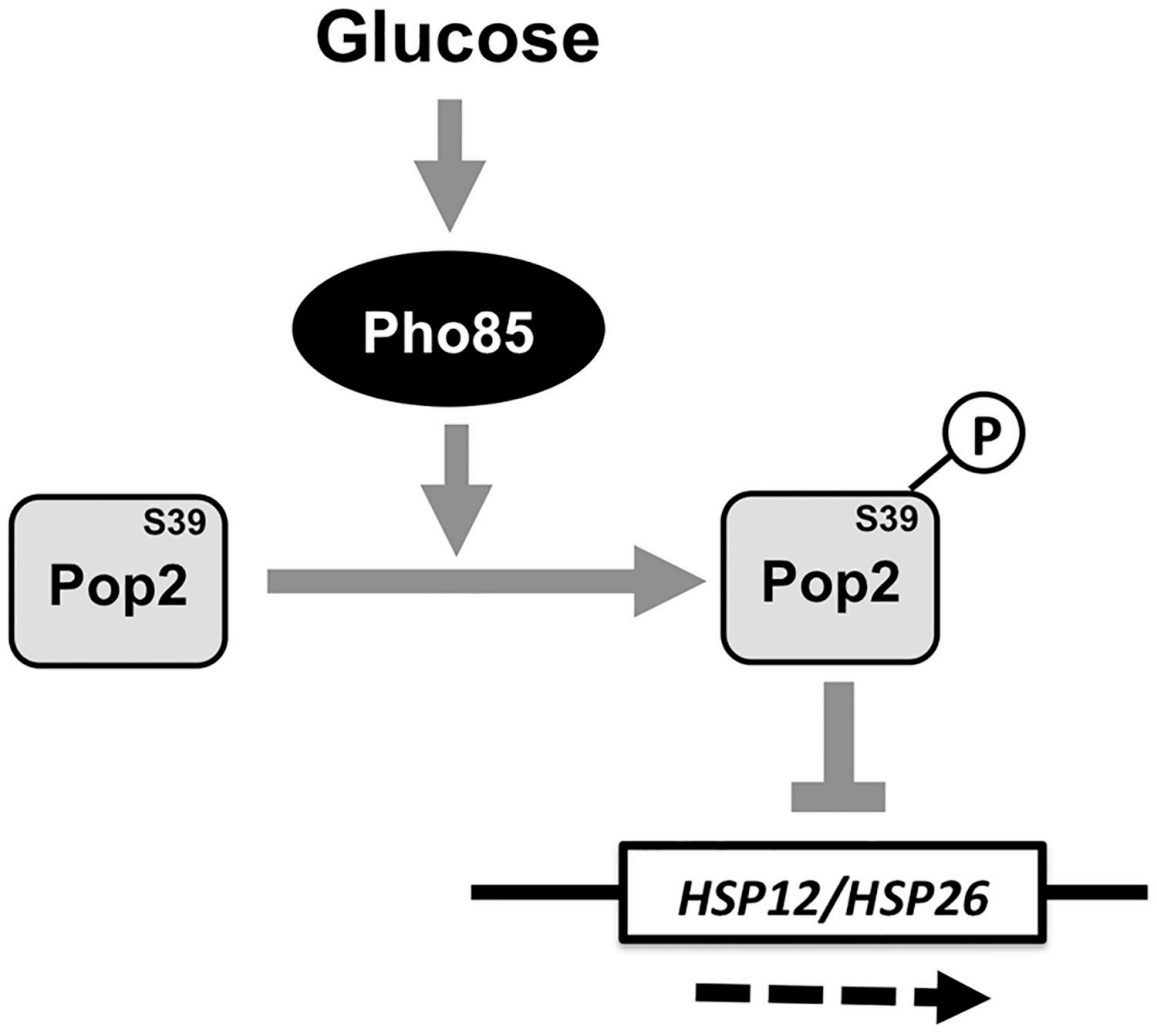
Proposed model of Pho85-dependet phosphorylation of Pop2 to repress the expression of stress response genes, *HSP12* and *HSP26*. In glucose medium, Pho85 is activated by glucose to phosphorylate Pop2 at S39. This phosphorylation regulates Pop2 function to repress the expression of stress response genes, *HSP12* and *HSP26*.

Taken together, we have shown that Pop2 is phosphorylated in a Pho85-dependent manner and this phosphorylation contributes to glucose repression of stress response genes, *HSP12* and *HSP26*. Based on the present data, Fig 8 shows our proposed model. Our study provides valuable insights the role of Pop2 in glucose repression of stress responses in yeast. The molecular mechanism how Pop2 regulates *HSP12* and *HSP26* mRNAs through S39 phosphorylation will be the focus of future studies.

## Supporting information

S1 Table Strains used in this study. (DOCX)

S2 Table Plasmids used in this study. (DOCX)

S3 Table *POP2* deletion induced the expression of stress response genes in YPD medium. (DOCX)

S1 File References. (DOCX)
