# Supplementary material for: Pop2 phosphorylation at S39 contributes to the glucose repression of stress response genes, HSP12 and HSP26
Source: PLoS One. 2019 Apr 11;14(4):e0215064. doi: 10.1371/journal.pone.0215064 (PMC6459547; doi:10.1371/journal.pone.0215064)
Supplement: S1 Table — (DOCX) [file pone.0215064.s001.docx]

| **S1 Table. Yeast strains used in this study.** | | | |
| --- | --- | --- | --- |
| No. | Strains | Genotype | Reference |
| 1 | 10B | *MAT*α *ade2 trp1 can1 leu2 his3 ura3 GAL psi+ HOp-ADE2-HO 3' UTR* | 36 |
| 2 | 10BD | *MAT***a***/MAT*α *ade2/ade2 trp1/trp1 can1/can1 leu2/leu2 his3/his3 ura3/ura3* | 36 |
| 3 | 10BD-p | *MAT***a***/MAT*α *ade2/ade2 trp1/trp1 can1/can1 leu2/leu2 his3/his3 ura3/ura3 POP2/pop2Δ::CgLEU2* | This study |
| 4 | p-1 | *MAT*α *ade2 trp1 can11 leu2 his3 ura3 pop2Δ::CgLEU2* | This study |
| 5 | 10BD-ph | *MAT***a***/MAT*α *ade2/ade2 trp1/trp1 can1/can1 leu2/leu2 his3/his3 ura3/ura3 PHO85/pho85Δ::CgHIS3* | This study |
| 6 | ph-1 | *MAT*α *ade2 trp1 can11 leu2 his3 ura3 pho85Δ::CgHIS3* | This study |
| 7 | snf | *MAT*α *ade2 trp1 can11 leu2 his3 ura3 snf1Δ::CgTRP1* | This study |
| 8 | 10BD-y | *MAT***a***/MAT*α *ade2/ade2 trp1/trp1 can1/can1 leu2/leu2 his3/his3 ura3/ura3 YAK1/yak1Δ::CgHIS3* | This study |
| 9 | y-1 | *MAT*α *ade2 trp1 can11 leu2 his3 ura3 yak1Δ::CgHIS3* | This study |
