# Supplementary material for: Pop2 phosphorylation at S39 contributes to the glucose repression of stress response genes, HSP12 and HSP26
Source: PLoS One. 2019 Apr 11;14(4):e0215064. doi: 10.1371/journal.pone.0215064 (PMC6459547; doi:10.1371/journal.pone.0215064)
Supplement: S2 Table — (DOCX) [file pone.0215064.s002.docx]

| **S2 Table. Plasmids used in this study.** | | | |
| --- | --- | --- | --- |
| **No.** | **Name** | **Relevant markers** | **Source or reference** |
| 1 | YCplac33 | *URA3, CEN-ARS* | 37 |
| 2 | YCplac33-POP2FLAG | *URA3, CEN-ARS, POP2FLAG* | This study |
| 3 | YCplac33-POP2SAFLAG | *URA3, CEN-ARS, POP2SAFLAG* | This study |
| 4 | YCplac33-POP2TAFLAG | *URA3, CEN-ARS, POP2TAFLAG* | This study |
| 5 | YCplac33-POP2SATAFLAG | *URA3, CEN-ARS, POP2SATAFLAG* | This study |
| 6 | pRS306 | *URA3* | 38 |
| 7 | pRS306-POP2FLAG | *URA3, POP2FLAG* | This study |
| 8 | pRS306-POP2SAFLAG | *URA3, POP2SAFLAG* | This study |
| 9 | pCgLEU2 | *C. glabrata LEU2 in pUC19* | 39 |
| 10 | pCgHIS3 | *C. glabrata HIS3 in pUC19* | 39 |
| 11 | pFA6a-kanMX6 | *kanMX6* | 18 |
