# Supplementary material for: Pop2 phosphorylation at S39 contributes to the glucose repression of stress response genes, HSP12 and HSP26
Source: PLoS One. 2019 Apr 11;14(4):e0215064. doi: 10.1371/journal.pone.0215064 (PMC6459547; doi:10.1371/journal.pone.0215064)
Supplement: S3 Table — (DOCX) [file pone.0215064.s003.docx]

**S3 Table. *POP2* deletion induced the expression of stress response genes in YPD medium.**

|  | **Wild-type** | ***pop2Δ*** |
| --- | --- | --- |
| *HSP12* mRNA level | 653 | 5719 |
| *HSP26* mRNA level | 101 | 3550 |
| *PIR3* mRNA level | 189 | 4030 |
| *ACT1* mRNA level | 10711 | 8922 |
| *PGK1* mRNA level | 10819 | 8871 |
