# Supplementary material for: Pop2 phosphorylation at S39 contributes to the glucose repression of stress response genes, HSP12 and HSP26
Source: PLoS One. 2019 Apr 11;14(4):e0215064. doi: 10.1371/journal.pone.0215064 (PMC6459547; doi:10.1371/journal.pone.0215064)
Supplement: S1 File — (DOCX) [file pone.0215064.s004.docx]

**Supporting Information**

**References**

36. Tadauchi T, Matsumoto K, Herskowitz I, Irie K. Post-transcriptional regulation through the *HO* 3′-UTR by Mpt5, a yeast homolog of Pumilio and FBF. The EMBO Journal. 2001;20(3):552-61. doi: 10.1093/emboj/20.3.552. PMID: 11157761

37. Gietz RD, Akio S. New yeast-Escherichia coli shuttle vectors constructed with in vitro mutagenized yeast genes lacking six-base pair restriction sites. Gene. 1988;74(2):527-34. doi: 10.1016/0378-1119(88)90185-0. PMID: 3073106

38. Sikorski RS, Hieter P. A system of shuttle vectors and yeast host strains designed for efficient manipulation of DNA in *Saccharomyces cerevisiae*. Genetics. 1989;122(1):19-27. PMID: 2659436

39. Sakumoto N, Mukai Y, Uchida K, Kouchi T, Kuwajima J, Nakagawa Y, et al. A series of protein phosphatase gene disruptants in *Saccharomyces cerevisiae*. Yeast. 1999;15(15):1669-79. doi: 10.1002/(SICI)1097-0061(199911)15:15<1669::AID-YEA480>3.0.CO;2-6. PMID: 10572263
